# Temperature-Induced Increase in Methane Release from Peat Bogs: A Mesocosm Experiment

**DOI:** 10.1371/journal.pone.0039614

**Published:** 2012-06-29

**Authors:** Julia F. van Winden, Gert-Jan Reichart, Niall P. McNamara, Albert Benthien, Jaap S. Sinninghe. Damsté

**Affiliations:** 1 Department of Geosciences, Utrecht University, Utrecht, The Netherlands; 2 Alfred-Wegener-Institut für Polar- und Meeresforschung, Bremerhaven, Germany; 3 Centre for Ecology and Hydrology, Lancaster Environment Centre, Lancaster, United Kingdom; 4 Department of Marine Organic Biogeochemistry, NIOZ Royal Netherlands Institute for Sea Research, Den Burg, The Netherlands; University of California Irvine, United States of America

## Abstract

Peat bogs are primarily situated at mid to high latitudes and future climatic change projections indicate that these areas may become increasingly wetter and warmer. Methane emissions from peat bogs are reduced by symbiotic methane oxidizing bacteria (methanotrophs). Higher temperatures and increasing water levels will enhance methane production, but also methane oxidation. To unravel the temperature effect on methane and carbon cycling, a set of mesocosm experiments were executed, where intact peat cores containing actively growing *Sphagnum* were incubated at 5, 10, 15, 20, and 25°C. After two months of incubation, methane flux measurements indicated that, at increasing temperatures, methanotrophs are not able to fully compensate for the increasing methane production by methanogens. Net methane fluxes showed a strong temperature-dependence, with higher methane fluxes at higher temperatures. After removal of *Sphagnum*, methane fluxes were higher, increasing with increasing temperature. This indicates that the methanotrophs associated with *Sphagnum* plants play an important role in limiting the net methane flux from peat. Methanotrophs appear to consume almost all methane transported through diffusion between 5 and 15°C. Still, even though methane consumption increased with increasing temperature, the higher fluxes from the methane producing microbes could not be balanced by methanotrophic activity. The efficiency of the *Sphagnum*-methanotroph consortium as a filter for methane escape thus decreases with increasing temperature. Whereas 98% of the produced methane is retained at 5°C, this drops to approximately 50% at 25°C. This implies that warming at the mid to high latitudes may be enhanced through increased methane release from peat bogs.

## Introduction

After remaining stable for almost a decade, methane concentrations in the atmosphere have started to rise again since 2007 [1]. Increasing emissions from the warming high northern latitude wetlands are probably responsible for this observed rise in methane [2]. This is important since methane is a potent greenhouse gas, having a potential impact at least 25 times that of CO_2_ [3]. Since the industrial revolution atmospheric methane concentrations increased as a consequence of changes in land use, agriculture and industrial activity [4]. Natural sources for atmospheric methane include wetlands and peatlands in the in the tropics and at mid-to high latitudes.

Peat bogs play an important role in the global carbon cycle. On the one hand they are the largest terrestrial carbon sink, on the other hand they are an important natural source of atmospheric methane, a potent greenhouse gas [5], [6]. Methane emissions from peat bogs, however, are strongly reduced by aerobic methane oxidizing bacteria (methanotrophs) [7], [8]. Future climatic change projections indicate that mid to high latitudes, especially Western Siberia with the largest peat bogs globally may become increasingly wetter and warmer [9]. It is therefore necessary to understand the influence of these environmental factors on methane cycling in peat bogs.

Methane oxidizing activity by methanotrophs strongly depends on temperature and local relative water level. A temperature increase from 10 to 20°C roughly resulted in a doubling in methane oxidation activity in *Sphagnum-*associated methanotrophs and also higher water levels resulted in higher methane oxidation rates [8]. On the other hand, methanogenic activity in peat bogs displays a strong correlation with water level and temperature [10], [11], suggesting that warming and increasing rainfall could lead to increased rates of methane generation. Here we study the balance of methane production and methane oxidation relative to in-situ water level, and investigate whether increased methane production as a consequence of increasing temperatures might be balanced by enhanced methanotrophic activity.

Our study was performed at Moorhouse Nature reserve (North Pennines, UK), an acidic ombrotrophic blanket bog incised with numerous gullies [12]. On the blanket, *Sphagnum* mosses (*S. capillifolium)* grow above the water level, while in waterlogged areas *Sphagnum* (*S. cuspidatum)* grows at or below the water level (nomenclature after Smith [13]). In order to establish the influence of water level on methane oxidation rates at the field site, *Sphagnum* mosses from different relative water levels were analysed for their potential methane oxidation rates. Also, methane production rates were determined for the top peat layers, by incubation in serum flask bottles. Subsequently, the influence of temperature on methane production and oxidation were evaluated by incubation of intact *Sphagnum* peat cores at various temperatures. After two months, methane fluxes from these peat cores were measured, with and without the presence of the methane-oxidizing *Sphagnum* layer.

## Results and Discussion

**Figure 1:**
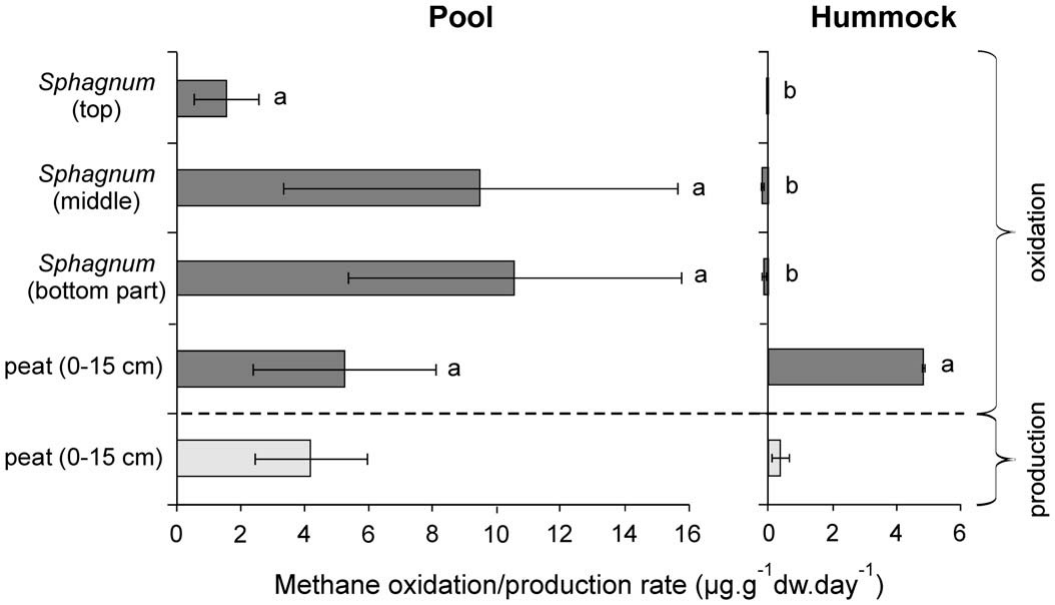
Potential methane oxidation rates (grey bars) and production rates (white bars). *Sphagnum* plants and peat from a pool-site and a hummock-site were analysed. *Sphagnum* plants were divided in three parts. Rates are expressed in µg.g dw^−1^.day^−1^ and are means of triplicate incubations ± s.d. Letters indicate statistically significant groups of data (*P*<0.05).

Highest potential methane oxidation rates were observed in pool-derived *S. cuspidatum*, which experiences relatively high water level, the pool-site (Fig. 1). This is in accordance with previous studies [8], [14], [15]. The highest methane potential methane oxidation rates were observed for the lower parts of *Sphagnum* plants from pools, although these values were not significantly different from the top part (*P*>0.05). Hummock *Sphagnum*, which grows above the water level, exhibited no methane oxidation (Fig. 1). Both peat horizons demonstrated methane oxidizing capacity (Fig. 1). Although pool-derived peat is situated well below the water level where oxygen is virtually absent, methanotrophs apparently quickly become active when oxygen is provided. The top part of the hummock-derived peat is situated just around the water level, providing a good position for methanotrophic bacteria along the methane gradient. Methane production rates were higher in pool-derived peat compared to hummock-peat (Fig. 1). Waterlogged areas are also the local hotspots for methane emissions in peat bogs [14]. In hummock-peat, organic matter degradation of *Sphagnum* largely takes place in the aerobic top layer (acrotelm), leaving less organic matter for anaerobic degradation processes [11]. The observed methane oxidation potential of hummock peat is more than sufficient to oxidize all produced methane. This balance is more critical in pool settings, suggesting that these pool settings are more susceptible to environmental change.

**Figure 2:**
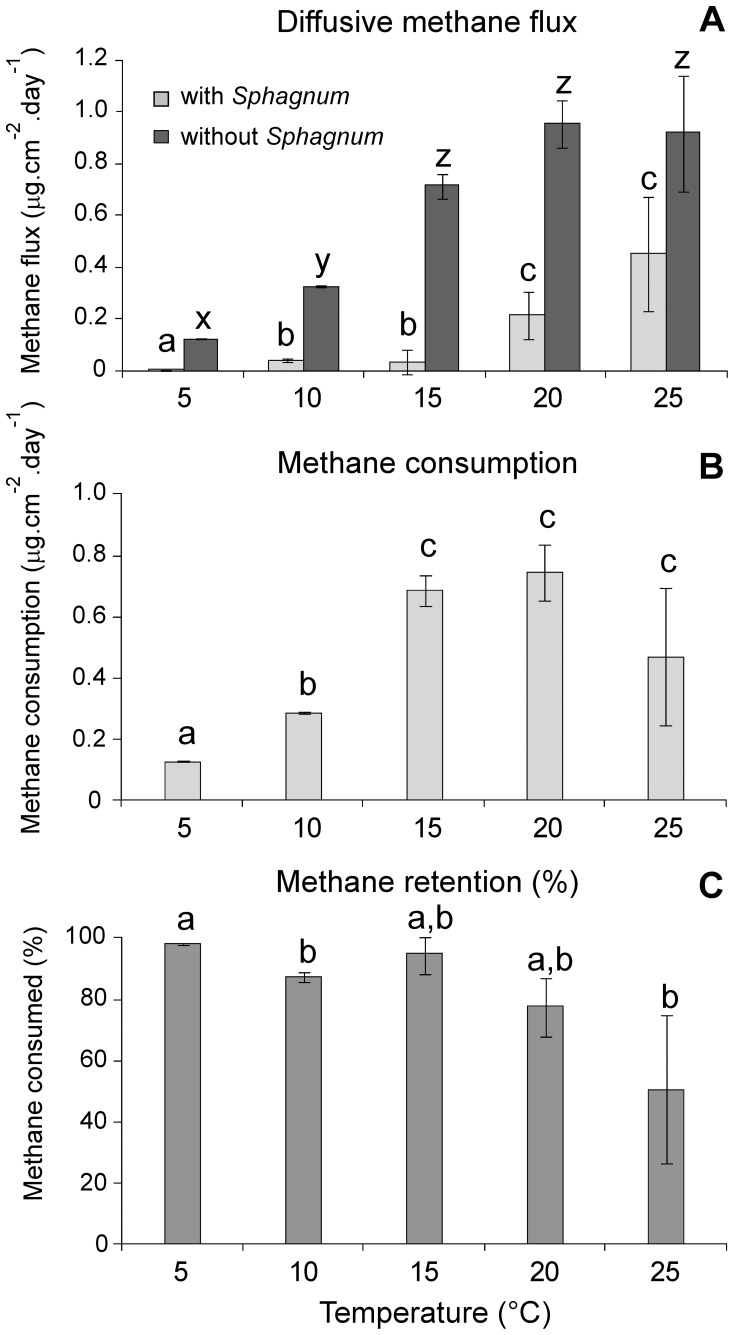
Methane cycling at different temperatures. A) Diffusive methane flux, with and without *Sphagnum*, B) methane consumption, the difference in methane flux before and after removal of *Sphagnum*, C) methane retention. Fluxes are measured on small peat cores after two months of incubation and values are expressed in µg.cm^−2^.day^−1^. Methane retention is expressed in % of the initial flux measured without *Sphagnum*. Values represent means of triplicate incubations ± s.d. Letters indicate statistically significant groups of data (*P*<0.05). Diffusive methane flux data with and without *Sphagnum* were not compared to each other.

Temperature is known to enhance bacterial methane oxidation as well as archaeal methane production [8], 10,11. The net effect of both these processes remains, however, unclear. To unravel the temperature effect on methane and carbon cycling, intact peat cores containing actively growing *Sphagnum* were incubated at 5, 10, 15, 20, and 25°C, where *Sphagnum* growth rates as well as methane fluxes were measured. Net methane fluxes showed a significant (*P*<0.05) and strong (Q10_10–20°C_ *= *5.2*)* temperature-dependence, with higher methane fluxes at higher temperatures (Fig. 2A). This suggests that the temperature-induced increase in methane production was higher than the increase in methane consumption. After removal of *Sphagnum*, methane fluxes were significantly (*P*<0.05) higher and increased with temperature (Q10_10–20°C_ *= *3.3) This indicates that the methanotrophs associated with *Sphagnum* plants play an important role in reducing the net methane flux from peat. Methane consumption was reconstructed by calculating the difference in the methane flux before and after the removal of *Sphagnum*. Methane consumption was significantly (*P*<0.05) different and increased with temperature (Q10_10–20°C_ *= *2.6), reaching maximum values around 20°C (Fig. 2B). This suggests that this is the optimum temperature for methanotrophs residing in peat bogs. Even though these measurement were only done for a limited number of replicates, they show a clear trend and are in line with previous results [8], [10], [11].

**Figure 3:**
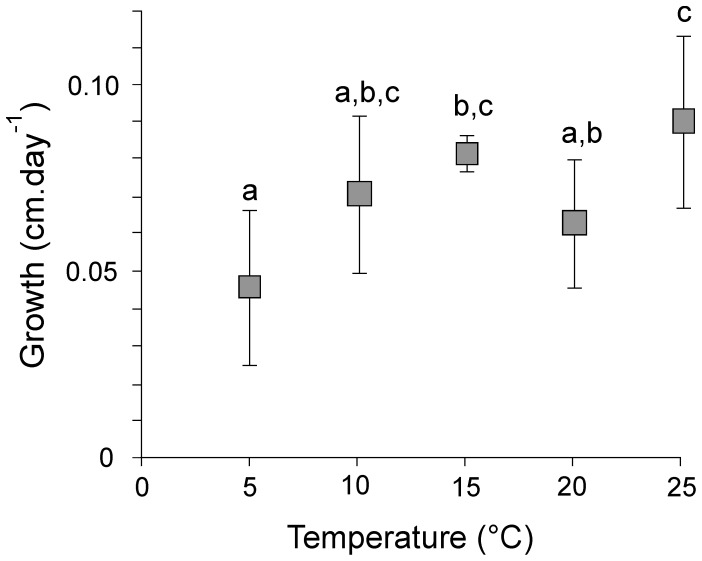
Growth rates of *Sphagnum* at different temperatures. Growth rates are measured after two months of incubation. Values are expressed in cm and represent means of four replicates ± s.d. Letters indicate statistically significant groups of data (*P*<0.05).

The efficiency of the *Sphagnum*-methanotroph consortium to act as a filter preventing the escape of methane appeared to be 90–100% in the lower temperature range (Fig. 2C). Methanotrophs appear to consume almost all methane transported through diffusion under these conditions. Methane retention showed a strong temperature-dependence beyond 15°C, dropping to only about 50% at 25°C (Fig. 2C). Even though methane consumption increased with increasing temperature, the higher fluxes from the methane-producing microbes could not be balanced by methanotrophic activity. Reduced solubility of methane with increasing temperature may be also in part responsible for the observed relationship. Growth rates of *Sphagnum* did significantly differ with temperature (P<0.05), with highest growth rates observed at the highest temperature (Fig. 3). Increasing CO_2_ assimilation in conjunction with increasing temperature potentially results in enhanced carbon storage.

Our results indicate that the *Sphagnum-*methanotroph consortium plays an important role in reducing methane emissions in peat bogs, potentially preventing the release of methane via diffusive transport up to 98%. Climate change projections indicate that mid- to high latitudes, where peat bogs are primarily situated, will become warmer as well as wetter [9]. Even though wetter conditions would increase methane oxidation rates, it would also enhance methane production rates, with most probably ultimately higher methane emissions. The effect of increasing methane emissions by increased wetness could be counteracted by enhanced carbon storage through peat bog growth [16]. Also higher temperatures could result in enhanced carbon storage, when *Sphagnum* growth rates increase with increasing temperatures. Nonetheless, methane fluxes increased with increasing temperature. Even though methane consumption increased with increasing temperature, methanotrophs appeared to be not able to fully compensate for the increased methane production, over the given time period. Methane retention dropped from approximately 98% at 5°C to only about 50% at 25°C. This may partially explain the recently observed rise in wetland methane emissions from mid- to high latitudes [1]. It is not expected that the northern peat bogs will experience a temperature increase of 25°C on average. However, the range of temperatures used for this study covers the range of temperatures expected during the period in which methane cycling plays an important role, the summer. The purpose of this study is to mechanistically understand the balance in methane production and oxidation with respect to temperature, and this study gives an indication into which direction the balance will tend to shift. A long-term consequence of global warming at mid- to high latitudes may also be a shift in the plant community towards vascular plants [17]. This would also result in higher methane emissions, as the oxidizing layer of the *Sphagnum*-methanotroph consortium will be lost in favour of vascular plants which act as conduits for the escape of methane. Hence, when mid- to high latitudes become increasingly warmer as well as wetter, peat bogs will most probably become a larger source for atmospheric methane, and therefore may act as a positive feedback to global rising temperatures.

## Materials and Methods

### Site Description

This study was performed in Moorhouse Nature Reserve, North Pennines, UK (54°41′38′’N, 2°22′40′’W). This setting has been described in detail elsewhere [12]. In short, Moorhouse is an acidic (pH 3.0 to 4.2) blanket bog, intersected by gullies. The vegetation on the blanket is typical for hummock peat, containing *Calluna vulgaris, Eriophorum vaginatum, Eriophorum angustifolium, Pleurozium schreberi* and *Sphagnum capillifolium*. In waterlogged areas, e.g. pools or gullies, vegetation was dominated by *Sphagnum cuspidatum*, *E. angustifolium* and *E. vaginatum*. The mean monthly temperature is approximately 10°C and average rainfall is high at 1900 mm per year.

Our research was performed using material collected from a pool site and a hummock site. The pool site is characterized by a wide gully with limited lateral flow and dominated by *S. cuspidatum,* with the top of the decomposing peat approximately 15 cm below the water table. The hummock site is dominated by *S. capillifolium* on blanket peat at approximately 10 m distance from the pool site, with water levels approximately 5 cm below the top of the capitula, as the conditions during sampling were relatively wet.

### Potential Methane Oxidation and Production Rates

*S. cuspidatum* and peat were sampled from the pool-site and *S. capillifolium* and peat were sampled from the hummock-site, after which the *Sphagnum* mosses were washed with demineralized water and sectioned into top, middle and lower parts, approximately 3 cm each. From both sites, peat was also sampled using a small spade, retrieving the top 15 cm. Prior to incubation, the peat was mixed. All samples (approximately 0.5 g dry weight) were incubated in triplicate in 120 ml serum vials and mixed with 40 ml demineralized water and capped with a gas-tight stopper. Incubations continued between 4 and 14 days.

For determining methane production potential vials were flushed with nitrogen and incubations were performed at room temperature (20°C) without shaking or stirring. To measure potential methane oxidation rates, 1 ml methane was added to each vial and kept under continuous shaking. Methane concentrations in the serum flasks were analysed daily at the CEH in Lancaster using a PerkinElmer (PerkinElmer, USA) Autosystem Gas Chromatograph (GC) fitted with a flame ionisation detector operating at 130°C. The GC contained a stainless steel Porapak Q 50–80 mesh column (length 2 m, outer diameter 3.17 mm), maintained at 100°C with a helium carrier gas flowing at 60 ml min^−1^. A calibration line was constructed by injection of a series of standards with different known concentrations. Potential methane oxidation and production were subsequently calculated using the initial linear rates. Reported rates are the average of three incubations. A student t-test was performed to analyse the differences at different depths.

### Mesocosm Experiments

Peat cores (length 50 cm, diameter 7 cm) were recovered from the pool-site. Perspex tubes were pushed into the peat until the core top was 5 cm above the water level, recovering about 30 cm of peat. The core was subsequently dug out by hand and capped at the bottom with a rubber stopper. The *S. cuspidatum* on top was sampled separately and added to the top water layer. The air temperature at the site was 12.5°C at the time of sampling, and the bog water had a temperature of 11.5°C.

The cores were incubated at 5, 10, 15, 20, and 25°C in incubators (Rumed™) (5, 10 and 20°C) and in climate rooms (15 and 25°C) at the AWI, Bremerhaven for two months. During the incubation water levels were kept at the top of the capitula of the *Sphagnum*, by adding bog water originating from the field site. The cores were transparent but the bottom 30 cm containing peat were covered with aluminium foil. For all experiments light level was set, at 65 µmol m^−2^ s^−1^, with a 24 h light-dark cycle similar to summer conditions (16 h light, 8 h dark). The *Sphagnum* was harvested after two months, when the photosynthetic capitulum (top 3 cm) was newly grown in all set-ups. Growth rates were calculated from the increment in height of the tops of the capitula since the start of the experiment. This was measured for four cores per experiment.

At the end of the mesocosm experiments methane fluxes were measured by closing the Perspex tubes with airtight caps equipped with rubber septa and obtaining headspace gas samples at several time intervals using a syringe and needle. After removal of the living *Sphagnum* layer, methane flux measurements were repeated. Methane concentrations were measured with a Thermo Finnigan Trace GC fitted with a flame ionisation detector operating at 300°C. The GC was equipped with a Restek PC 6031 packed column (length 2 m, 2 mm internal diameter), maintained at 100°C, using helium as the carrier gas flowing at 7 ml min^−1^. Methane fluxes were calculated from the average of the four measurements, and standard deviations were calculated for the replicates.

### Statistical Analyses

Pairwise comparisons were made for methane oxidation rates, methane fluxes with and without *Sphagnum*, methane consumption, methane retention, and *Sphagnum* growth at different temperature using ANOVA. Groups that showed significant differences were assigned different letters. Correlations between incubation temperatures and methane fluxes with and without *Sphagnum*, methane consumption, methane retention, and *Sphagnum* growth were also tested by ANOVA. Statistical analyses were performed in SPSS 18.0.
